# Functional Foods as Vehicles for Bioactive Compounds: Chemical and Nutritional Perspectives on Health and Disease Prevention

**DOI:** 10.3390/ijms27052293

**Published:** 2026-02-28

**Authors:** Rita Paola Debri, Antonino De Lorenzo, Raffaele Conte, Gianfranco Peluso

**Affiliations:** 1 Faculty of Medicine and Surgery, Saint Camillus International University of Health Sciences, Via di Sant’Alessandro 8, 00131 Rome, Italy; rpaola.debri@unicamillus.org (R.P.D.); gianfranco.peluso@unicamillus.org (G.P.); 2 Department of Biomedicine and Prevention, University of Rome “Tor Vergata”, Via Montpellier 1, 00133 Rome, Italy; delorenzo@uniroma2.it; 3 Research Institute on Terrestrial Ecosystems (IRET)-CNR, Via Pietro Castellino 111, 80131 Naples, Italy; 4 National Biodiversity Future Center (NBFC), 90133 Palermo, Italy

**Keywords:** functional foods, bioactive compounds, polyphenols, antioxidant activity, gut microbiota modulation, chronic disease prevention, nutritional biochemistry

## Abstract

Functional foods are a central paradigm in modern nutrition science, acting as effective vehicles for the delivery of bioactive compounds that link conventional nutrition and preventive medicine. Beyond their basic nutritional role, these foods are specifically designed or naturally enriched to convey biologically active constituents capable of modulating physiological functions and reducing the risk of chronic diseases, thereby supporting long-term health maintenance. The chemical composition of functional foods—including polyphenols, phytosterols, vitamins and dietary fibers—underlies their capacity to act as matrices that protect, transport, and enhance the bioavailability of bioactive molecules. This review provides an integrated nutritional perspective on functional foods, with particular emphasis on their role as delivery systems for health-promoting compounds. The molecular mechanisms by which food bioactives interact with cellular and molecular targets, regulate oxidative stress and inflammation, and modulate metabolic and immune pathways are critically discussed. Special attention is devoted to redox-active bioactives, the structural diversity and bioavailability of polyphenols, the cholesterol-lowering properties of phytosterols, the physiological relevance of fat- and water-soluble vitamins, and the complex interactions between functional foods, gut microbiota, prebiotics, probiotics, and dietary fibers. Overall, this review aims to provide a comprehensive scientific framework for understanding how functional foods can be strategically engineered and utilized as bioactive compound vehicles in health promotion and disease prevention.

## 1. Introduction

The relationship between diet and health has been recognized since antiquity, with early medical traditions already acknowledging the role of food in maintaining physiological balance and preventing illness. However, contemporary nutritional science has moved far beyond the traditional concept of food as a mere source of energy and essential nutrients required for survival [1]. Advances in biochemistry, molecular biology, and systems physiology have revealed that food components can actively modulate metabolic pathways, cellular signaling, gene expression, and immune responses [2]. In recent decades, the rapidly increasing prevalence of chronic non-communicable diseases—such as cardiovascular disorders, type 2 diabetes mellitus, obesity, metabolic syndrome, neurodegenerative diseases, and several forms of cancer—has intensified scientific interest in dietary strategies aimed not only at disease treatment but also at prevention and health promotion. Then, the concept of functional foods considerably evolved, gaining substantial relevance at scientific, clinical, and industrial levels [3].

Functional foods are generally defined as foods that, when consumed as part of a habitual diet and in physiologically relevant amounts, exert beneficial effects on one or more target functions in the body beyond basic nutritional roles, thereby improving overall health status or reducing the risk of disease development [4]. Unlike nutraceuticals or dietary supplements, which are typically formulated in concentrated or pharmaceutical-like forms (e.g., capsules, tablets, or powders), functional foods retain their conventional food matrix and are seamlessly integrated into daily dietary patterns [4]. This distinction is particularly important, as it highlights the potential of functional foods to exert sustained, long-term effects at the population level while minimizing issues related to compliance, safety, and excessive intake.

From a biochemical perspective, the functionality of these foods is intrinsically linked to the presence of specific bioactive compounds [5]. These include polyphenols, carotenoids, phytosterols, vitamins, bioactive peptides, and structurally diverse carbohydrates such as oligosaccharides and dietary fibers. These compounds display remarkable structural heterogeneity, encompassing variations in molecular weight, polarity, redox potential, and functional groups, which ultimately influence their reactivity and biological behavior [5]. Importantly, their health effects depend not only on intrinsic chemical structure but also on extrinsic factors such as food matrix composition, processing and storage conditions, bioaccessibility during digestion, intestinal absorption, metabolic transformation, tissue distribution, and interactions with host enzymes, receptors, and signaling networks [6].

One of the central biochemical themes linking functional foods to human health is the regulation of oxidative balance [7]. Oxidative stress, defined as an imbalance between the generation of reactive oxygen and nitrogen species (ROS and RNS) and the capacity of endogenous antioxidant defense systems, plays a pivotal role in the initiation and progression of numerous chronic and degenerative diseases. Persistent oxidative stress can lead to lipid peroxidation, protein oxidation, DNA damage, and dysregulation of redox-sensitive signaling pathways [8]. Functional foods provide a rich and diverse source of exogenous antioxidants that complement endogenous defenses such as superoxide dismutase, catalase, glutathione peroxidase, and non-enzymatic antioxidants [9]. The chemistry of these dietary antioxidants, including their redox mechanisms, metal-chelating properties, radical-scavenging activity, and synergistic interactions, is therefore important in understanding their protective effects at the cellular and systemic levels. In parallel, growing attention has been directed toward the gut microbiota as a critical mediator of the relationship between diet and health. The human gastrointestinal tract is a highly complex and dynamic microbial ecosystem that actively participates in nutrient metabolism, immune modulation, maintenance of intestinal barrier integrity, and the regulation of inflammatory responses [10]. Functional foods can influence both the composition and metabolic activity of the gut microbiota through the intake of prebiotics, probiotics, and fermentable dietary fibers. These interactions lead to the production of biologically active metabolites, such as short-chain fatty acids, which exert local and systemic effects on glucose and lipid metabolism, immune function, and neuroendocrine signaling [11].

Moreover, the nutritional relevance of functional foods extends to lipid metabolism and vitamin homeostasis. Phytosterols, due to their structural similarity to cholesterol, can competitively inhibit intestinal cholesterol absorption, contributing to the reduction in plasma LDL cholesterol levels and associated cardiovascular risk [12]. Fat-soluble and water-soluble vitamins, beyond their classical role in preventing deficiency-related diseases, participate in redox regulation, immune competence, epigenetic modulation, cellular differentiation, and signal transduction pathways [13,14]. Their stability during processing, bioavailability, metabolic fate, and interactions with other dietary constituents are critical determinants of their functional efficacy [15].

This review aims to provide an overview of the nutritional properties of functional foods and their relevance as vehicles for bioactive compounds, with particular emphasis on how functional foods themselves act as complex food-based delivery matrices that protect bioactive compounds from chemical and enzymatic degradation, modulate their release during gastrointestinal digestion, enhance bioaccessibility and intestinal absorption, and influence their metabolic transformation and tissue distribution.

## 2. Oxidative Stress and Redox Homeostasis

### 2.1. Molecular Basis and Biological Significance of Oxidative Stress

Oxidative stress is a central biochemical concept for understanding the role of functional foods in human health promotion. Oxidative stress arises when the production of reactive oxygen species (ROS) exceeds the capacity of antioxidant defense systems, leading to a disruption of redox homeostasis [16]. Redox balance is essential for normal cellular function, as tightly regulated oxidation–reduction reactions are involved in mitochondrial energy production, signal transduction, gene expression, immune responses, and cellular adaptation to environmental stimuli [16].

Reactive oxygen species comprise both free radical and non-radical oxygen-derived molecules, including superoxide anion (O_2_•^−^), hydrogen peroxide (H_2_O_2_), hydroxyl radical (•OH), singlet oxygen (^1^O_2_), and peroxyl radicals (ROO•). Free radicals are characterized by the presence of one or more unpaired electrons in their outer orbital, a feature that confers high chemical instability and reactivity [17]. In biological systems, this reactivity drives electron abstraction from nearby molecules in an attempt to achieve energetic stability, thereby initiating chain reactions that can propagate oxidative damage across cellular structures [17].

Importantly, ROS are not intrinsically harmful. Under physiological conditions, they are continuously generated at low concentrations and fulfill essential biological functions [18]. Controlled ROS production participates in host defense mechanisms, such as the respiratory burst of immune cells, and acts as a secondary messenger in redox-sensitive signaling pathways that regulate cell proliferation, differentiation, apoptosis, and adaptive stress responses [18]. Oxidative stress develops when ROS generation becomes excessive, prolonged, or poorly controlled, overwhelming antioxidant systems and shifting ROS signaling from physiological regulation to pathological damage [19].

In this context, functional foods acquire relevance. Through the intake of bioactive compounds such as vitamins, carotenoids, polyphenols, and essential trace elements, these foods can modulate endogenous antioxidant defenses and contribute to the maintenance or restoration of cellular redox equilibrium.

### 2.2. Endogenous and Exogenous Sources of Reactive Oxygen Species

Reactive oxygen species originate from both endogenous metabolic processes and exogenous environmental exposures. The principal endogenous source of ROS is mitochondrial oxidative phosphorylation. During electron transfer along the respiratory chain, a small fraction of electrons leak from complexes I and III, leading to the partial reduction in molecular oxygen and the formation of superoxide anions [20].

Additional endogenous ROS are generated through enzymatic reactions involving oxidases, oxygenases, and peroxidases, as well as during inflammatory responses. Activated neutrophils and macrophages deliberately produce large amounts of ROS as part of antimicrobial defense mechanisms [21]. Furthermore, hepatic xenobiotic metabolism, particularly phase I reactions mediated by the cytochrome P450 enzyme system, generates ROS as by-products [22]. Ischemia–reperfusion events constitute another important source of oxidative stress, as the sudden reintroduction of oxygen into previously hypoxic tissues results in acute bursts of ROS production [23].

Alongside endogenous metabolism, numerous exogenous factors significantly contribute to oxidative burden. Environmental exposures such as ultraviolet and ionizing radiation, air pollution, particulate matter, tobacco smoke, heavy metals, pesticides, and industrial chemicals promote ROS formation [24]. Lifestyle-related factors, including unbalanced diets, excessive alcohol consumption, psychological stress, and exposure to certain pharmacological agents and chemotherapeutics, further exacerbate oxidative stress [24].

### 2.3. Endogenous Antioxidant Systems

Redox homeostasis in human cells is maintained by a tightly regulated endogenous antioxidant network composed of enzymatic and non-enzymatic components that operate in a coordinated and compartment-specific manner. Rather than acting as isolated scavengers, these systems function as an integrated redox-control machinery that limits excessive ROS accumulation while preserving physiologically relevant redox signaling [25].

Among the enzymatic antioxidants, superoxide dismutases (SODs) represent the primary defense against superoxide anion (O_2_•^−^), one of the earliest ROS generated during mitochondrial respiration and cellular metabolism. By catalyzing its dismutation into hydrogen peroxide (H_2_O_2_) and molecular oxygen, SODs prevent superoxide-mediated damage and redirect redox flux toward more controllable intermediates [25]. The existence of distinct SOD isoforms, localized in the cytosol (Cu/Zn-SOD), mitochondria (Mn-SOD), and extracellular space, ensures spatially resolved protection consistent with the compartmentalized nature of ROS generation [26].

Hydrogen peroxide, although less reactive than superoxide, plays a dual role as both a signaling molecule and a potential precursor of highly cytotoxic radicals. Its intracellular concentration is therefore strictly controlled by catalase and glutathione peroxidases (GPxs). Catalase, predominantly localized in peroxisomes, exhibits exceptionally high turnover rates, enabling the rapid detoxification of large H_2_O_2_ fluxes, particularly under conditions of acute oxidative challenge [27]. In parallel, selenium-dependent GPxs reduce hydrogen peroxide and lipid hydroperoxides using reduced glutathione (GSH) as an electron donor, providing fine-tuned redox regulation in cellular compartments where catalase activity is limited [28]. Non-enzymatic endogenous antioxidants complement enzymatic defenses by acting predominantly as preventive and buffering agents. Metal-binding proteins, including transferrin, ferritin, ceruloplasmin, and lactoferrin, exert a crucial protective role by limiting the bioavailability of redox-active iron and copper ions [29]. Through metal sequestration, these proteins effectively suppress radical-generating reactions and represent a first line of defense against oxidative chain initiation [29].

In addition, low-molecular weight metabolites contribute substantially to systemic antioxidant capacity. Bilirubin, derived from heme catabolism, has emerged as a physiologically relevant antioxidant capable of efficiently scavenging peroxyl radicals and inhibiting lipid peroxidation at circulating concentrations [30]. Similarly, uric acid, the end product of purine metabolism in humans, displays significant antioxidant activity through both radical scavenging and metal-chelating mechanisms. Its abundance in plasma and epithelial lining fluids underscores its role as a key redox buffer, particularly at the interface between endogenous metabolism and environmental oxidant exposure [31]. While endogenous antioxidant systems provide the primary and indispensable framework for redox control, their efficiency can be modulated and supported by exogenous antioxidants supplied through the diet. Bioactive compounds present in functional foods do not simply act as direct radical scavengers, but interact with endogenous defense pathways, influence redox-sensitive signaling, and contribute to the maintenance of oxidative balance under conditions of increased metabolic, environmental, or inflammatory stress.

## 3. Functional Foods as Vehicle of Antioxidants

### 3.1. Polyphenol-Rich Functional Foods

Dietary polyphenols are widely recognized as key functional food components contributing to health maintenance and the prevention of chronic diseases. Epidemiological evidence associates a high consumption of polyphenol-rich foods with reduced incidence of cardiovascular diseases, type 2 diabetes, neurodegenerative disorders, and certain cancers [32,33]. However, the strength of these associations varies substantially across populations and study designs, with some cohort studies reporting modest or non-significant effects after adjustment for overall dietary quality and lifestyle confounders.

Large prospective cohort studies, including those conducted within Mediterranean and plant-based dietary patterns, have demonstrated inverse correlations between total polyphenol intake and all-cause mortality, highlighting the clinical relevance of these compounds beyond their antioxidant capacity. For instance, high polyphenol intake (>800–1000 mg/day) has been associated with approximately 20–30% lower all-cause mortality in large European cohorts, although heterogeneity among intake assessment methods remains a major limitation [34,35]. Importantly, the protective effects attributed to polyphenols cannot be ascribed solely to isolated molecules, but rather to their consumption within functional foods, where the complex food matrix plays a decisive role in modulating their stability, bioaccessibility, and biological efficacy [36]. Functional foods rich in polyphenols act as protective dietary platforms by delivering these compounds in combination with fibers, lipids, proteins, vitamins, and minerals that influence their release during digestion, intestinal absorption, and metabolic transformation [37]. Nevertheless, human bioavailability studies indicate that only 5–10% of ingested parent polyphenols typically reach systemic circulation in native form, underscoring the importance of matrix effects and gut microbiota metabolism. This matrix-mediated delivery enhances the capacity of polyphenols to exert biological effects, including the attenuation of oxidative stress, modulation of inflammatory signaling pathways, improvement of endothelial function, and regulation of glucose and lipid metabolism. Furthermore, polyphenol-rich functional foods contribute to the maintenance of redox homeostasis not only through direct radical-scavenging activity but also by upregulating endogenous antioxidant defenses and modulating redox-sensitive transcription factors. However, some controlled trials report limited changes in systemic oxidative stress biomarkers, suggesting that indirect signaling mechanisms rather than classical antioxidant activity may predominate in vivo [38].

Flavonoids are plant-derived pigments belonging to the polyphenol class, widely distributed in fruits, vegetables, and plant-based foods, where they contribute to coloration and serve important protective functions in plants. In human nutrition, flavonoids are recognized for their antioxidant, anti-inflammatory, and vasoprotective properties. Flavonoid-rich functional foods represent the most extensively investigated examples of polyphenol-based functional foods. Regular consumption of flavonol-containing foods, such as apples, onions, and leafy vegetables, has been associated with improved endothelial function, reduced blood pressure, and lower cardiovascular risk, with quercetin frequently identified as a major contributor to these effects [39]. Meta-analyses of randomized trials indicate that quercetin supplementation at doses of 500–1000 mg/day can reduce systolic blood pressure by approximately 3–5 mmHg, although results are inconsistent in normotensive subjects and short-duration interventions. Intervention studies have shown that quercetin supplementation or quercetin-rich diets can attenuate markers of oxidative stress and inflammation, partly through the modulation of nitric oxide bioavailability and inhibition of lipid peroxidation [40]. Notably, interindividual variability linked to gut microbiota composition has emerged as a key determinant of responsiveness.

Flavanols from cocoa and green tea provide another well-documented example of polyphenols acting as functional foods in disease prevention. Cocoa flavanol intake has been repeatedly linked to improvements in flow-mediated dilation, arterial stiffness, and cognitive performance. Acute and short-term trials report FMD improvements of ~1–2 percentage points following cocoa flavanol doses of 200–900 mg/day, a magnitude considered clinically meaningful for cardiovascular risk reduction. These findings support a protective role against cardiovascular and age-related cognitive decline, highlighting cocoa’s role as a functional food in which the cocoa butter lipid matrix, soluble and insoluble fibers, and endogenous polyphenol–protein complexes synergistically protect flavanols from degradation and facilitate their sustained release and bioefficacy [41]. Similarly, green tea catechins, particularly epigallocatechin-3-gallate (EGCG), have been associated with a reduced risk of cardiovascular disease, obesity, and metabolic syndrome, as well as with chemopreventive effects in hormone-related and gastrointestinal cancers. Clinical trials typically employ EGCG doses ranging from 150 to 800 mg/day, with modest but significant reductions in LDL cholesterol (≈5–10%) reported in several meta-analyses. These benefits are attributed to combined antioxidant, anti-inflammatory, and metabolic regulatory actions that are further reinforced by the green tea matrix acting as a functional carrier, in which catechins form non-covalent complexes with dietary macromolecules, reducing EGCG degradation, improving bioaccessibility, and enabling prolonged antioxidant and anti-inflammatory effects [42]. Conversely, high-dose green tea extracts have occasionally been associated with hepatotoxicity, highlighting the need to distinguish between whole-food consumption and concentrated supplements. Anthocyanin-rich foods, including berries, red grapes, and purple vegetables, are increasingly recognized as functional foods with broad preventive potential. Observational studies and randomized controlled trials have reported associations between anthocyanin intake and a reduced risk of myocardial infarction, improved insulin sensitivity, and enhanced lipid profiles [43]. Pooled analyses suggest that anthocyanin intakes of ~50–320 mg/day are associated with improvements in insulin sensitivity indices and modest increases in HDL cholesterol, although effect sizes remain variable across trials. These effects are attributed not only to the intrinsic bioactivity of anthocyanins but also to the functional food matrix of berries and red grapes, where organic acids, dietary fibers, and co-occurring polyphenols stabilize anthocyanins, protect them from pH- and enzyme-mediated degradation, and modulate gut microbiota-dependent metabolism, thereby enhancing their bioaccessibility and cardiometabolic protective actions [43]. Blueberries, blackberries, and blackcurrants, in particular, have been shown to improve vascular function and cognitive performance in both adult and elderly populations, suggesting a role in the prevention of cardiovascular and neurodegenerative diseases [44]. Nevertheless, several randomized trials report neutral cognitive outcomes, especially in short-term interventions or low-dose protocols, indicating that duration and metabolite exposure thresholds are critical determinants of efficacy. These benefits are further supported by berries acting as functional foods, where the interaction between anthocyanins, fiber, and micronutrients preserves polyphenol bioactivity, facilitates blood–brain barrier-relevant metabolite formation, and supports endothelial and neuronal resilience [44].

Isoflavones derived from soy products represent a distinct class of polyphenols with functional relevance, especially in hormone-related health outcomes. Populations with a high habitual intake of soy-based foods exhibit a lower incidence of osteoporosis, cardiovascular disease, and certain hormone-dependent cancers. Clinical and experimental studies indicate that isoflavones such as genistein and daidzein exert antioxidant and anti-inflammatory effects while modulating estrogen receptor signaling, contributing to their protective role in postmenopausal health [45]. Randomized trials using 40–80 mg/day of soy isoflavones report modest improvements in bone turnover markers and small reductions in LDL cholesterol (~3–6%), although effects on fracture risk remain inconclusive. These effects are further supported by soy acting as a functional food matrix, in which isoflavones are naturally present as glycoside conjugates and interact with proteins, fibers, and lipids, protecting them from premature degradation and modulating intestinal hydrolysis and absorption, thereby enhancing their estrogenic, antioxidant, and anti-inflammatory efficacy [45]. Citrus fruits, including oranges and lemons, represent a distinctive class of functional foods in which flavanones such as hesperidin and naringenin contribute to anti-inflammatory activity, vascular protection, and improved lipid homeostasis. Clinical studies using hesperidin doses of 500–1000 mg/day have reported reductions in inflammatory markers (e.g., CRP) and modest improvements in endothelial function, although findings are not uniformly consistent. In citrus, these compounds are structurally integrated within a pectin-rich and acidic matrix that influences their release kinetics during digestion, favors enzymatic conversion into absorbable forms, and supports colonic biotransformation into metabolites with enhanced biological relevance. Through this food-specific architecture, citrus fruits enable a sustained exposure to flavanone-derived bioactives, ultimately supporting endothelial integrity, modulation of lipid metabolism, and attenuation of low-grade inflammation [46]. The cited examples are summarized in Table 1.

### 3.2. Functional Foods Rich in Phytosterols and Polyunsaturated Fatty Acids

Phytosterols and polyunsaturated fatty acids (PUFAs) represent two major classes of lipophilic bioactive compounds whose health benefits are closely linked to their incorporation within functional food matrices. Epidemiological and clinical evidence consistently demonstrates that diets rich in phytosterol- and PUFA-containing foods are associated with improved lipid profiles, reduced cardiovascular risk, and modulation of the inflammatory and metabolic pathways [47]. However, the magnitude of these benefits varies considerably depending on baseline cardiometabolic status, background diet, and formulation of the functional food vehicle, indicating that not all enriched products provide equivalent clinical efficacy.

The biological efficacy of these compounds depends not only on their intrinsic chemical properties but also on the capacity of functional foods to act as effective delivery vehicles that ensure their stability, bioaccessibility, and physiological availability. Functional foods provide a lipid-based matrix that facilitates the solubilization, protection, and controlled release of phytosterols and PUFAs during gastrointestinal digestion [48]. Bioaccessibility studies indicate that the micellar incorporation efficiency of phytosterols can vary by more than twofold depending on the fat content and emulsification state of the food matrix, highlighting an important but often overlooked source of variability. Indeed, due to their structural similarity to cholesterol, phytosterols competitively inhibit intestinal cholesterol absorption by displacing cholesterol from mixed micelles, thereby reducing plasma LDL cholesterol levels. The food matrix plays a critical role in this process, as the presence of dietary fats, emulsifiers, and phospholipids enhances micellar incorporation and intestinal uptake, optimizing the cholesterol-lowering efficacy of phytosterols [48]. Meta-analyses of randomized controlled trials consistently show that daily phytosterol intakes of 1.5–3.0 g reduce LDL cholesterol by approximately 7–12%, with a dose–response plateau observed above ~3 g/day [49]. Similarly, functional foods enriched in PUFAs—particularly omega-3 and omega-6 fatty acids—act as vehicles that promote efficient digestion, absorption, and tissue incorporation of these highly unsaturated lipids, which are otherwise susceptible to oxidative degradation [50]. Nevertheless, oxidation during processing and storage remains a critical limitation for PUFA-enriched products and may attenuate their biological efficacy if not properly controlled.

Beyond lipid lowering, PUFA-rich functional foods exert protective effects through modulation of membrane composition, regulation of eicosanoid synthesis, and generation of specialized pro-resolving lipid mediators involved in the control of inflammation and immune responses [51]. The structural organization of the food matrix influences PUFA bioavailability and metabolic fate, affecting their incorporation into cell membranes and their conversion into bioactive metabolites [51]. Stable isotope studies suggest that incorporation of EPA and DHA into plasma phospholipids typically reaches 2–4% of total fatty acids after several weeks of intake, although interindividual variability is substantial [52]. Moreover, functional foods can enhance the oxidative stability of PUFAs by co-delivering antioxidants such as tocopherols and polyphenols, thereby preserving their biological activity throughout digestion and metabolism [53]. Among the lipid-derived bioactive compounds, phytosterols and PUFAs, particularly omega-3 and omega-6 fatty acids, have been extensively investigated as functional food components for the modulation of cardiometabolic risk. Phytosterols, including β-sitosterol, campesterol, and stigmasterol, are naturally occurring plant sterols structurally analogous to cholesterol and are abundant in vegetable oils (e.g., corn, soybean, rapeseed oil), whole grains, legumes, nuts, and seeds. Their cholesterol-lowering properties have been consistently demonstrated in both experimental and clinical settings, positioning phytosterols among the most robustly validated functional food ingredients [54]. However, their impact on hard cardiovascular endpoints (e.g., myocardial infarction, stroke) remains insufficiently demonstrated, representing an important translational gap. Mechanistically, phytosterols reduce intestinal cholesterol absorption by competing with dietary and biliary cholesterol for incorporation into mixed micelles formed by bile salts. This competitive displacement limits cholesterol solubilization and subsequent uptake by enterocytes [54]. Similarly, human intervention studies using phytosterol-enriched foods—such as margarines, yogurts, milk-based beverages, and cereal products—have repeatedly shown reductions in plasma LDL cholesterol ranging from 7% to 12% with daily intakes of 1.5–3.0 g of phytosterols [55]. Notably, randomized controlled trials have reported significant LDL-lowering effects following the consumption of phytosterol-fortified spreads and dairy products without detrimental effects on HDL cholesterol or triglyceride levels, supporting their application in primary cardiovascular prevention [55]. Conversely, concerns have been raised regarding the potential accumulation of circulating phytosterols in rare genetic conditions (e.g., sitosterolemia), although this appears clinically negligible in the general population [56]. At the cellular level, phytosterols entering enterocytes are inefficiently esterified and are actively transported back into the intestinal lumen via ATP-binding cassette transporters ABCG5 and ABCG8. This efflux system limits systemic exposure to phytosterols while enhancing fecal sterol excretion. The consequent reduction in intracellular cholesterol content stimulates hepatic LDL receptor expression, promoting the increased clearance of circulating LDL particles [55]. Given the central role of oxidized LDL in foam cell formation and atherogenesis, dietary phytosterol intake indirectly attenuates vascular inflammation and plaque development. Functional foods enriched with phytosterols have therefore been incorporated into dietary guidelines for individuals with mild to moderate hypercholesterolemia as a non-pharmacological strategy to reduce cardiovascular risk [57]. Nuts, such as almonds and pistachios, are recognized as functional foods due to their high content of phytosterols, which have been consistently associated with lowering LDL cholesterol, improving overall lipid profiles, and promoting cardioprotective effects. Clinical feeding trials indicate LDL reductions of approximately 5–10% with daily nut intakes of 30–60 g, although the relative contribution of phytosterols versus unsaturated fats and fiber remains difficult to disentangle [58]. The protective action of nuts is mediated not only by the intrinsic activity of phytosterols but also by the complex food matrix, which includes unsaturated fats, fibers, and proteins. This matrix enhances the solubility and intestinal incorporation of phytosterols, reduces their degradation during digestion, and modulates their absorption and systemic availability. By facilitating the effective delivery of bioactive phytosterols, nuts contribute to the regulation of cholesterol metabolism, reduction in atherogenic lipoproteins, and overall cardiovascular health [59].

In parallel, omega-3 and omega-6 polyunsaturated fatty acids exert profound effects on membrane composition, inflammatory signaling, and lipid mediator biosynthesis, making them key targets in functional nutrition. Omega-6 fatty acids, predominantly linoleic acid derived from vegetable oils such as sunflower, corn, and soybean oil, serve as precursors to arachidonic acid and downstream pro-inflammatory eicosanoids. However, recent meta-analyses indicate that moderate linoleic acid intake is not consistently associated with increased cardiovascular risk, challenging earlier assumptions of uniformly pro-inflammatory effects [60]. For example, vegetable oils rich in ω-6 fatty acids, such as sunflower and corn oil, are important functional food components due to their high content of linoleic acid, which serves as a precursor for arachidonic acid and a variety of bioactive eicosanoids that modulate inflammatory responses. The protective action of these oils is influenced by their natural matrix, composed of triglycerides, phospholipids, tocopherols, and minor bioactive compounds, which enhances the stability and bioavailability of linoleic acid during digestion. This matrix facilitates the efficient incorporation of ω-6 fatty acids into cell membranes, allowing for balanced eicosanoid synthesis and the regulation of immune and inflammatory pathways [61]. However, excessive omega-6 intake, characteristic of Western dietary patterns, has been associated with chronic low-grade inflammation, endothelial dysfunction, and increased susceptibility to cardiometabolic disorders [62]. This apparent discrepancy likely reflects the importance of overall dietary context and the omega-6/omega-3 balance rather than absolute omega-6 intake alone.

Conversely, omega-3 fatty acids—particularly eicosapentaenoic acid (EPA) and docosahexaenoic acid (DHA)—are abundant in fatty fish (e.g., salmon, sardines, mackerel), fish oils, and algae-based products, and are widely recognized as functional food components with anti-inflammatory and cardioprotective properties [63]. Epidemiological studies, including large prospective cohort analyses, have consistently linked regular fish consumption with reduced cardiovascular mortality [63]. Prospective cohort analyses typically report 15–30% lower cardiovascular mortality among individuals consuming ≥2 fish servings per week. The protective effects of these foods are not only due to the intrinsic bioactivity of EPA and DHA but are also supported by the fish matrix, which is rich in phospholipids, proteins, and other lipids that enhance the stability, absorption, and systemic delivery of omega-3 fatty acids [63]. Clinical trials have also demonstrated that omega-3-enriched functional foods, such as fortified dairy products, eggs, and bakery items, can lower plasma triglycerides, improve endothelial function, and reduce markers of systemic inflammation [64]. Triglyceride reductions of 15–30% are commonly observed with EPA+DHA intakes of 2–4 g/day, whereas lower doses in fortified foods often produce more modest effects. At the molecular level, EPA and DHA are incorporated into cell membrane phospholipids, displacing arachidonic acid and altering membrane microdomain organization and receptor signaling. They also serve as precursors to specialized pro-resolving mediators, including resolvins, protectins, and maresins, which actively orchestrate the resolution phase of inflammation. Functional foods enriched with omega-3 fatty acids have been shown to modulate platelet aggregation, reduce vascular oxidative stress, and enhance plaque stability, thereby lowering the risk of acute cardiovascular events [64].

Importantly, the dietary omega-6/omega-3 ratio has emerged as a critical determinant of inflammatory tone. Functional food strategies aimed at correcting the excessively high omega-6/omega-3 ratio typical of Western diets—through increased omega-3 intake and partial substitution of omega-6-rich oils—have demonstrated beneficial effects on metabolic health, insulin sensitivity, and inflammatory biomarkers. Intervention studies targeting ratios below ~4:1 generally report improvements in inflammatory biomarkers and insulin sensitivity, although an optimal universal ratio has not been definitively established [64,65]. For example, replacement of conventional cooking oils with omega-3-enriched oils or the consumption of omega-3-fortified foods has been associated with improvements in lipid profiles and inflammatory status in both healthy individuals and populations at metabolic risk [65]. A summary of the cited examples is available in Table 2.

### 3.3. Vitamin-Rich Functional Foods

Vitamins constitute an essential class of micronutrients whose biological functions extend far beyond the prevention of classical deficiency syndromes, playing critical roles in redox homeostasis, immune competence, cellular differentiation, epigenetic regulation, and signal transduction pathways [66]. The health impact of vitamins is strongly influenced by the capacity of functional foods to act as effective delivery vehicles, ensuring their chemical stability, bioaccessibility, and physiological availability within the human body. Unlike isolated vitamin supplements, vitamins consumed through functional foods are embedded within complex food matrices that modulate their release during digestion, protect them from degradation, and facilitate their interaction with other dietary components [67]. This distinction is clinically relevant, as food-based vitamin delivery often results in slower absorption kinetics and potentially improved metabolic utilization compared with high-dose supplements.

Functional foods provide a particularly advantageous delivery platform for fat-soluble vitamins (A, D, E, and K), whose absorption is highly dependent on the presence of dietary lipids, emulsifying agents, and bile-mediated micellar formation. The lipid architecture of the food matrix enhances solubilization and intestinal uptake, thereby improving bioavailability and tissue distribution [68,69]. For example, human bioavailability studies indicate that the co-ingestion of ≥10–15 g of dietary fat can increase carotenoid and vitamin D absorption by two- to threefold compared with low-fat conditions [70]. Additionally, functional foods can protect fat-soluble vitamins from oxidative and photo-induced degradation by co-delivering antioxidants such as polyphenols and tocopherols, preserving their biological activity throughout processing, storage, and digestion [71].

Water-soluble vitamins, including the B-complex vitamins and vitamin C, exhibit saturable transport mechanisms. Very high oral doses may not proportionally increase systemic availability, highlighting the importance of physiologically relevant intake ranges. Then, these substances benefit from food-based delivery systems. The structural organization of functional foods influences their stability, gastrointestinal release kinetics, and interaction with specific intestinal transporters. Moreover, the co-occurrence of vitamins with bioactive peptides, minerals, and fermentable fibers can modulate absorption efficiency and metabolic utilization, supporting synergistic physiological effects [72].

Vitamins, both fat- and water-soluble, are essential dietary components that modulate oxidative stress and preserve cellular redox homeostasis, playing a critical role in the prevention of chronic diseases such as atherosclerosis, type 2 diabetes, neurodegeneration, and inflammatory disorders [73].

Among the soluble dietary lipids, vitamin E, primarily α-tocopherol, is abundant in vegetable oils, nuts, seeds, and whole grains, and has been shown in epidemiological studies and RCTs to reduce plasma lipid peroxidation, protect LDL from oxidative modification, and improve endothelial function in hyperlipidemic and diabetic subjects [74]. Meta-analyses of supplementation trials reported reductions in circulating malondialdehyde and oxidized LDL of approximately 10–20% with doses ranging from 200 to 800 IU/day, although cardiovascular outcome benefits remain inconsistent. The protective effects of these foods are enhanced by their functional food matrices, which include lipids, fibers, and proteins that stabilize α-tocopherol during digestion, promote its intestinal absorption, and support its systemic delivery [74]. Carotenoids, including β-carotene, lutein, and zeaxanthin, present in carrots, spinach, kale, peppers, and apricots, have been associated with lower oxidative biomarkers, improved macular pigment density, and reduced cardiovascular risk in large cohort studies and intervention trials [75]. Randomized trials typically report increases in macular pigment optical density of 5–20% after 3–12 months of lutein/zeaxanthin intake (10–20 mg/day), although effects on visual performance are variable. In these foods, the combination of fibers, lipids, and naturally co-occurring phytochemicals creates a supportive environment that protects carotenoids from digestive breakdown, facilitates their absorption in the gut, and maintains their biological activity [75]. Vitamin D, obtained from fatty fish, fortified dairy, and sun-induced synthesis, modulates oxidative stress indirectly through the suppression of NADPH oxidase activity, enhancement in glutathione peroxidase expression, and reduction in pro-inflammatory cytokines [76]. Clinical trials using vitamin D doses of 800–2000 IU/day consistently raised the serum 25 (OH)D concentrations by ~10–30 ng/mL, yet effects on cardiometabolic endpoints remained modest or inconsistent in many populations. When obtained from foods, its protective effects are further supported by the functional food matrix—lipids, proteins, and other micronutrients present in fish and dairy enhance vitamin D stability, facilitate its intestinal absorption, and promote its incorporation into circulation [76]. Vitamin K, found in leafy greens, broccoli, and fermented foods, contributes to vascular protection and lowers oxidative stress markers, partly by preserving endothelial nitric oxide bioavailability [77]. Observational studies link higher dietary vitamin K intake with reduced vascular calcification and ~10–20% lower cardiovascular risk, although randomized evidence remains limited. By interacting with lipids, fibers, and coexisting phytochemicals, vitamin K in these foods is stabilized and more effectively absorbed, allowing sustained delivery to the endothelium and optimized vascular and antioxidant protection [77]. Among the water-soluble vitamins, vitamin C from citrus fruits, kiwi, strawberries, peppers, and berries directly scavenges reactive oxygen species, regenerates vitamin E, improves endothelial function, and lowers inflammatory markers [78]. Controlled trials generally report modest reductions in CRP (≈10–25%) and small improvements in endothelial function at intakes of 200–1000 mg/day, but effects on major cardiovascular outcomes are less consistent. The effectiveness of vitamin C is further supported by the natural composition of these foods, where sugars, organic acids, fibers, and coexisting phytochemicals create a protective environment that shields it from degradation, enhances its absorption, and prolongs its activity in the body [78]. B-vitamins, including folate, B6, and B12, contribute indirectly to oxidative stress regulation by controlling homocysteine metabolism and supporting one-carbon pathways, with folate-rich foods such as legumes, leafy greens, and fortified grains shown to reduce homocysteine and oxidative biomarkers in both healthy and at-risk populations [79]. Supplementation trials typically demonstrate homocysteine reductions of 15–30%, yet large cardiovascular outcome studies have not consistently shown parallel reductions in clinical events, indicating a complex relationship between biomarker modification and disease risk. The bioactivity of folate is enhanced by the complex food matrix, where fibers, proteins, and naturally co-occurring micronutrients help protect folate from degradation, support its controlled release during digestion, and facilitate efficient intestinal absorption [79]. Similarly, animal-derived foods such as meat, fish, poultry, eggs, and dairy are primary sources of vitamin B12 (cobalamin), a cofactor essential for homocysteine metabolism, DNA synthesis, and the indirect reduction in oxidative stress [80]. Beyond the intrinsic activity of B12, the natural food matrix plays a key role in its effectiveness: proteins, fats, and other micronutrients in these foods help protect cobalamin from degradation in the digestive tract, facilitate its release during enzymatic digestion, and support its uptake in the ileum [80]. Additionally, vitamin B6, mainly in the form of pyridoxal-5′-phosphate, is found in meat, poultry, fish, legumes, nuts, and cereals, and acts as a coenzyme in amino acid metabolism, reducing homocysteine and serving as an antioxidant cofactor [81]. Typical dietary intakes of 1.3–2.0 mg/day are sufficient for metabolic functions, and supraphysiological supplementation has not consistently demonstrated additional antioxidant benefit in controlled trials. Like B12, the effectiveness of B6 is supported by the surrounding food matrix, where proteins, fibers, and lipids protect the vitamin during digestion, modulate its release, and enhance intestinal absorption [81]. Inositol, a vitamin-like polyol abundant in whole grains, nuts, and brewer’s yeast, has demonstrated in preclinical and small clinical studies the ability to reduce oxidative stress via the modulation of Nrf2 and PI3K/Akt pathways while also improving insulin sensitivity and metabolic control [82]. Small randomized trials (typically 2–4 g/day myo-inositol) report improvements in insulin sensitivity indices and reductions in fasting glucose of ~5–10%, although large confirmatory studies are still lacking. Within these foods, inositol is embedded in a matrix of fibers, proteins, and other micronutrients that work together to preserve its stability, facilitate its transport across the intestinal barrier, and prolong its bioactivity [82]. Collectively, this evidence is summarized in Table 3.

## 4. Functional Food Action on Human Microbiota

The human microbiota is a complex and dynamic consortium of bacteria, archaea, viruses, and fungi residing on multiple body surfaces, functions as a metabolic and immunological organ essential for human health [83]. In the gut, a nutrient-rich and relatively neutral environment, commensal microbes such as *Bacteroides*, *Lactobacillus*, *Bifidobacterium*, and *Escherichia coli* participate in the fermentation of non-digestible carbohydrates into short-chain fatty acids (SCFAs) like acetate, propionate, and butyrate, which serve as both energy substrates for colonocytes and as signaling molecules that regulate host metabolic and immune functions [84]. SCFAs enhance epithelial barrier integrity, suppress pro-inflammatory cytokine production, and promote regulatory T-cell differentiation by inhibiting histone deacetylases and activating G protein-coupled receptors, thereby establishing a critical link between microbial metabolism and host immune homeostasis [85]. Dysbiosis, defined by reduced microbial diversity and depleted beneficial taxa, is implicated in the pathogenesis of autoimmune diseases such as rheumatoid arthritis, type 1 diabetes, and inflammatory bowel disease through immune dysregulation, increased intestinal permeability, and altered metabolite signaling [86].

Functional foods serve as structured dietary vehicles that transport microbiota-accessible substrates—including dietary fibers, resistant starches, polyphenols, and fermented components—into the gastrointestinal tract, where they selectively influence microbial composition and metabolic activity [87]. The food matrix plays a decisive role in determining the spatial and temporal availability of these substrates, modulating their fermentation kinetics and shaping microbial cross-feeding interactions within the gut ecosystem [88]. Through the delivery of prebiotics, probiotics, and synbiotic combinations, functional foods promote the growth and activity of beneficial microbial taxa while suppressing dysbiosis-associated microorganisms. Fermentable fibers and complex carbohydrates reach the colon largely intact, where they are metabolized by commensal bacteria into short-chain fatty acids (SCFAs) such as acetate, propionate, and butyrate [89]. These metabolites exert effects on host physiology, including the regulation of glucose and lipid metabolism, reinforcement of intestinal barrier integrity, modulation of immune and inflammatory responses, and communication with neuroendocrine pathways via the gut–brain axis [90]. Importantly, recent meta-analyses indicate that the magnitude of microbiota modulation by functional foods is highly heterogeneous across studies, with reported increases in fecal butyrate ranging from approximately 15% to over 60% depending on substrate type, dose (typically 3–15 g/day for fermentable fibers), and intervention duration (4–12 weeks). This variability underscores the need for standardized dosing frameworks and careful interpretation of cross-study comparisons [91].

Moreover, functional foods can facilitate the targeted delivery and survival of probiotic microorganisms by providing protective matrices that enhance viability during processing, storage, and gastrointestinal transit. Encapsulation within food structures, buffering against gastric acidity, and co-delivery with fermentable substrates improve colonization efficiency and functional persistence [92]. Fermented foods and probiotic interventions offer ways to modulate microbiota composition and function: for example, kefir consumption has been shown to increase the abundance of lactate-producing and SCFA-associated bacteria such as *Bifidobacterium breve* and *Blautia* in healthy adults, with concomitant enhancement of butyrate pathways and potentially improved gut health [93]. In controlled human trials, kefir intake (typically 200–400 mL/day for 4–8 weeks) has been associated with modest but statistically significant increases in fecal SCFA concentrations (≈10–25%), although some studies report no significant shifts in alpha diversity, highlighting interindividual variability and potential ceiling effects in healthy populations [94]. These protective effects are supported by the functional matrix of kefir, in which live microorganisms, peptides, bioactive lipids, and exopolysaccharides act synergistically to stabilize probiotic viability, enhance colonization and metabolic activity, and promote the production of SCFAs and other bioactive metabolites [93]. Kombucha ingestion has been associated with reduced pro-inflammatory taxa and increased beneficial genera such as *Lactobacillus* and *Mucispirillum*, resulting in improved metabolic and liver health outcomes in rodents [95], and modest enrichment of SCFA-producing taxa like *Weizmannia coagulans* in controlled human settings [96]. Nevertheless, translational relevance remains debated: rodent studies often report pronounced metabolic improvements (e.g., 20–40% reductions in hepatic lipid accumulation), whereas human trials generally show smaller or non-significant metabolic effects, likely reflecting differences in dose normalization, microbiota baseline composition, and study duration. The beverage’s protective effects are reinforced by its complex matrix of live microbes, polyphenols, and fermentation-derived metabolites, which preserve microbial activity, promote SCFA synthesis, and support antioxidant defenses [96]. Miso, a traditional fermented soybean paste, contains a diverse consortium of beneficial microorganisms, including *Lactobacillus*, *Enterococcus*, and *Bifidobacterium*, and has been associated with antioxidant activity, support of digestive enzyme function, immune modulation, and cardiovascular protection [97]. However, epidemiological data are partly conflicting, as some population studies link high miso intake with elevated sodium exposure, which may offset cardiovascular benefits in salt-sensitive individuals. Quantitative risk–benefit assessment therefore remains necessary when recommending habitual consumption. These effects are underpinned by miso’s functional food matrix, which arises from the fermentation of soy proteins and carbohydrates into bioactive peptides, amino acids, and microbial metabolites. This matrix preserves microbial viability, enhances enzymatic activity in the gastrointestinal tract, and promotes the production of antioxidant and anti-inflammatory compounds while also facilitating interactions with the gut microbiota [97,98]. Sauerkraut, a fermented cabbage product, is rich in lactic acid bacteria such as *Lactobacillus plantarum* and *Leuconostoc* species and has been associated with increased bioavailability of vitamins C and K, improved nutrient absorption, and modulation of the gut microbiota [99]. Intervention studies suggest that daily consumption (≈50–100 g/day) can increase fecal lactic acid bacteria counts by roughly 0.5–1.5 log units, although effects on clinical endpoints such as inflammatory markers remain inconsistent across trials. The protective effects of sauerkraut are driven by its functional food matrix, which is shaped by the lactic fermentation of cabbage fibers and phytochemicals. This process enhances vitamin stability, generates organic acids and bioactive metabolites, and promotes the survival and activity of beneficial microbes in the gastrointestinal tract [99]. In addition to the actions of fermented foods containing live probiotics, prebiotics such as inulin and fructooligosaccharides act as selectively fermentable substrates that nourish beneficial gut microorganisms and enhance their metabolic activity [100]. Dose–response analyses indicate that prebiotic supplementation in the range of 5–10 g/day typically increases *Bifidobacterium* abundance by 1–2 log units, although gastrointestinal tolerance (e.g., bloating) becomes more frequent above ~15 g/day, representing a practical upper limit for many individuals. On this basis, synbiotic interventions combining probiotics and prebiotics demonstrate enhanced microbial fermentation and elevated SCFA production, supporting metabolic regulation and reduction in harmful metabolites linked to inflammation and obesity. For example, synbiotic yogurt, formulated by combining probiotic strains such as *Lactobacillus rhamnosus* and *Bifidobacterium lactis* with prebiotic fibers including fructooligosaccharides (FOS) or inulin, has been shown to promote synergistic enhancement of short-chain fatty acid (SCFA) production, improved metabolic profiles, and protection of gut barrier integrity [101]. Randomized controlled trials report improvements in HOMA-IR of approximately 8–20% and modest reductions in circulating CRP (≈5–15%) after 6–12 weeks of synbiotic yogurt consumption, although not all studies reached statistical significance, reflecting heterogeneity in strain selection, viable counts (10^7^–10^10^ CFU/day), and host metabolic status. Within the yogurt functional matrix, the dairy protein–lipid network preserves probiotic viability and supports their survival through the gastrointestinal tract while fructooligosaccharides act as selectively fermentable substrates that stimulate the growth and metabolic activity of beneficial bacteria and downstream SCFA-producing taxa [101]. Collectively, while the preponderance of evidence supports a beneficial role of functional foods in microbiota modulation, the field is characterized by substantial heterogeneity in study design, microbial endpoints, and clinical outcomes. Future well-powered, strain-specific, and dose-standardized human trials are required to resolve current inconsistencies and define clinically meaningful effect thresholds. Table 4 summarizes the specific probiotic/prebiotic interventions with health outcomes.

## 5. Optimizing Bioactive Stability and Efficacy in Functional Foods

The effectiveness of functional foods as health-promoting agents is inherently tied to the chemical and physical stability, bioaccessibility, and systemic bioavailability of their constituent bioactive compounds. Many bioactives are chemically fragile and highly susceptible to degradation during food processing, storage, and gastrointestinal transit, conditions characterized by exposure to heat, oxygen, light, moisture, and pH fluctuations, which can cause oxidative breakdown, isomerization, hydrolysis, or interaction with other food components that reduce their biological activity and therapeutic efficacy [102]. To overcome these intrinsic stability issues, the food matrix itself plays a pivotal role in modulating the fate of bioactives during digestion and absorption. Interactions with proteins, lipids, polysaccharides, and micronutrients can sequester bioactives, limit their release, or alter their chemical form, substantially reducing the fraction that becomes bioaccessible during gastrointestinal digestion [103]. Additionally, the food matrix’s macronutrient composition and physical structure (e.g., fiber content, food particle size, and processing state) can dramatically influence the release kinetics of compounds from different food systems, underscoring the matrix’s critical influence on bioaccessibility [103].

In recent years, many advanced delivery and stabilization strategies capable of preserving, protecting, and enhancing the functional performance of bioactive compounds were developed. One of the most transformative approaches is nanoencapsulation, wherein bioactives are entrapped within nanometer-scale carriers constructed from food-grade lipids, proteins, polysaccharides, or synthetic polymers. By isolating sensitive molecules from environmental stressors, nanoencapsulation can markedly improve their physicochemical stability, storage life, and performance during digestion while facilitating enhanced dispersion and interaction with biological membranes [104]. Among the nanoencapsulation systems, nanoliposomes have emerged as flexible vehicles capable of accommodating both hydrophilic and lipophilic bioactives due to their phospholipid bilayer architecture. Nanoliposomes protect encapsulated molecules from degradation and offer controlled release properties that can be tuned through surface modification or composition adjustments, thereby enhancing stability and targeted delivery within the gastrointestinal tract. For instance, encapsulation of vitamins and antioxidant polyphenols into liposomal structures has been shown to protect these compounds from oxidative and enzymatic degradation while facilitating improved cellular uptake in vitro [105]. Liposomal vitamin C, for example, exhibits significantly enhanced stability and prolonged bioactivity compared with free ascorbic acid due to protection from oxidative catalysts and gastric degradation [106]. Liposomal formulations also improve intestinal permeability by promoting fusion with mucosal membranes and facilitating transcellular uptake [106].

Nanoemulsions represent another major class of delivery systems, particularly useful for lipophilic bioactives such as carotenoids, curcumin, and omega-3 fatty acids. These systems consist of oil-in-water droplets on the order of 20–200 nm, stabilized by food-grade emulsifiers, which provide high surface area and kinetic stability against coalescence [107]. Nanoemulsions have demonstrated their ability to protect sensitive compounds against isomerization and degradation during storage and gastrointestinal processing [108]. For example, curcumin nanoemulsions formulated with medium-chain triglycerides and protein stabilizers achieved bioaccessibility levels of 74–79% versus 1.7% for free curcumin, showcasing the dramatic enhancements possible through nanoemulsion technology [109]. Lipid-based nanoparticles such as solid lipid nanoparticles (SLNs) and nanostructured lipid carriers (NLCs) provide additional advantages for lipophilic compounds. By constructing a solid lipid matrix that remains solid at room and physiological temperatures, SLNs and NLCs confer rigid protective environments that reduce oxidative degradation and volatility of encapsulated bioactives; they are particularly adept at stabilizing carotenoids, vitamins, and essential oils against degradation and oxidation during processing and storage, thus improving their retention in functional food products. For example, omega-3 fatty acids and vitamin D encapsulated in SLNs have shown significantly improved resistance to oxidative stress and enhanced stability under storage, suggesting their potential for use in fortified foods such as dairy and beverage products [110]. In addition to lipid approaches, biopolymer-based nanoparticles—constructed from proteins and polysaccharides such as β-lactoglobulin, chitosan, alginate, pectin, and composite protein–polysaccharide matrices—offer “green” delivery platforms with excellent biocompatibility and regulatory acceptance. These carriers enhance bioactive stability by forming robust physical barriers and can be engineered to provide targeted release profiles through responsive changes to pH or ionic conditions encountered along the digestive tract. Protein–polysaccharide complexes, for instance, have been used to stabilize polyphenols (e.g., chlorogenic acid, gallocatechin gallate) during in vitro digestion, improving their gastric stability and accessibility compared with free forms [111].

Gum Arabic-coated nanostructures have likewise been shown to stabilize anthocyanins and enhance their cellular uptake and antioxidant activity. Such biopolymer systems also reduce undesirable interactions with the food matrix, improving solubility and functional efficacy without compromising sensory properties [112].

Polysaccharide carriers such as starch, maltodextrin, alginate, pectin, and inulin have demonstrated utility as micro- and nanocarrier wall materials for polyphenols and pigments, creating protective barriers that improve encapsulation efficiency (up to ~90%) and enhance bioaccessibility and functional performance in simulated digestion models. These materials can be designed to interact favorably with both hydrophilic and hydrophobic compounds, supporting their integration into complex food systems without adversely affecting texture or palatability. By entrapping polyphenols in a polysaccharide matrix, for example, controlled release throughout gastrointestinal transit is facilitated, improving their interaction with intestinal tissues and transport into systemic circulation [113]. Beyond traditional encapsulation approaches, advanced fabrication technologies such as electrospinning, electrospraying, microfluidic particle fabrication, and co-encapsulation strategies are expanding the possibilities for functional food design. Electrospinning and electrospraying produce ultra-fine polymer fibers or particles that can encapsulate bioactives in amorphous states, enhancing solubility and dissolution rates for poorly soluble compounds like curcumin or lycopene [114].

In parallel with encapsulation and nanodelivery, valorization of agro-industrial by-products have become integral to functional food development. Materials such as hazelnut skin, fruit pomace, cereal bran, and whey represent concentrated sources of polyphenols, fibers, vitamins, and peptides that can be extracted and stabilized for use in fortified foods. Using green solvent extraction techniques (e.g., ethanol or natural deep eutectic solvents) to recover bioactive fractions from food waste not only minimizes environmental impact but also yields nutraceutical ingredients with preserved functional properties suitable for incorporation into delivery systems. For example, hazelnut cuticle extracts high in catechins and quercetin have been fortified into plant-based beverages with high bioaccessibility, illustrating how valorized by-products can be transformed into bioefficacious ingredients [115].

Collectively, these technological advances not only enhances the functional impact of bioactive compounds but also supports the health benefits of sustainable production in modern food systems. The discussion is summarized in Table 5.

## 6. Conclusions

This review underscores the role of functional foods as a vehicle for bioactive compounds, providing a link between composition, nutritional function, and health promotion. The concept of food has shifted from being seen only as a source of energy to being recognized as a biologically active system that can influence physiological functions, support homeostasis, and interact with the body’s defense mechanisms. Indeed, functional foods act as complex delivery systems capable of transporting polyphenols, carotenoids, phytosterols, vitamins, bioactive peptides, fibers, and microbiota-modulating components to target sites within the body. Their effectiveness relies not only on the intrinsic properties of these bioactive molecules but also on the structural and physicochemical characteristics of the food matrix, which influence stability, gastrointestinal release, absorption, microbial metabolism, and systemic bioavailability. A recurring theme in the health-promoting potential of functional foods is the regulation of oxidative stress. Polyphenols, carotenoids and water- and fat-soluble vitamins all contribute to redox homeostasis. Importantly, the stability and bioaccessibility of these molecules are critical determinants of their in vivo efficacy. Several studies have repeatedly demonstrated that processing, storage, and gastrointestinal conditions can markedly reduce the bioactive potential of foods, highlighting the need of functional food and advanced formulation strategies. Parallel to antioxidant activity, the modulation of the gut microbiota has emerged as a critical mechanism through which functional foods exert systemic benefits. Prebiotics, probiotics, and fermentable fibers act synergistically to shape microbial communities, promoting beneficial taxa such as Lactobacillus and Bifidobacterium while suppressing potentially pathogenic populations. Fermented foods, including kefir, miso, kombucha, and sauerkraut, provide both live microbial strains and bioactive metabolites, illustrating how traditional dietary patterns naturally integrate microbiota-targeted interventions. The interplay between microbiota, prebiotic fibers, and bioactive compounds further highlights the need to consider the food matrix, digestion dynamics, and bioaccessibility in evaluating the functional impact of these foods. Finally, sustainability considerations increasingly intersect with functional food development, reinforcing the importance of valorizing by-products. Despite these advances, several challenges remain in translating compositional richness into measurable health outcomes. The chemical lability of bioactive molecules, interactions with complex food matrices, variable digestive kinetics, and interindividual differences in microbiota composition all contribute to heterogeneous responses. Therefore, rigorous evaluation of bioaccessibility, intestinal absorption, systemic bioavailability and bioefficacy is essential to bridge the gap between in vitro findings, nutritional composition data, and clinical efficacy. Multidisciplinary approaches integrating food chemistry, biotechnology, nutritional sciences, microbiology, and materials science are central to this effort. In conclusion, functional foods represent the base of modern nutrition, moving from caloric adequacy toward proactive modulation of health and disease risk. The integration of bioactive compounds, fibers, probiotics, and prebiotics, combined with advanced delivery technologies and sustainable sourcing, ensures that these foods can provide tangible, clinically relevant benefits. Functional foods, when effectively formulated and strategically integrated into daily dietary patterns, hold the potential to transform nutrition from a passive source of sustenance into an active instrument of health promotion and disease prevention.

## Figures and Tables

**Table 1:** Representative examples of functional foods as vehicles of dietary polyphenols.

Food Source	Main Polyphenols/Flavonoids	Main Biological Actions	Reference
Apples, onions, berries	Quercetin, kaempferol (flavonols)	Antioxidant and anti-inflammatory effects; improved endothelial function; reduced LDL oxidation and blood pressure	[39,40]
Cocoa, dark chocolate	Epicatechin, procyanidins (flavanols)	Improved vascular function; enhanced nitric oxide bioavailability; cognitive and cardiovascular benefits	[41]
Green tea	Epigallocatechin-3-gallate (EGCG), catechins (flavanols)	Modulation of oxidative stress and inflammation; cardiometabolic protection; chemopreventive effects	[42]
Red wine, grapes	Resveratrol, flavanols, anthocyanins	Cardiovascular protection; modulation of redox signaling; anti-inflammatory effects	[43]
Blueberries, blackberries, red grapes	Anthocyanins (cyanidin-, and delphinidin-derivatives)	Antioxidant and anti-inflammatory activity; improved insulin sensitivity; neuro- and cardioprotection	[44]
Soy and soy products	Genistein, daidzein (isoflavones)	Phytoestrogenic activity; antioxidant effects; bone, cardiovascular and hormonal health	[45]
Citrus fruits (oranges, lemons)	Hesperidin, naringenin (flavanones)	Anti-inflammatory and vasoprotective effects; improved lipid metabolism	[46]

Legend: LDL—Low-Density Lipoprotein; EGCG—Epigallocatechin-3-gallate; Flavonols—A class of flavonoids including quercetin and kaempferol; Flavanols—A class of flavonoids including catechins and procyanidins; Flavanones—A class of flavonoids including hesperidin and naringenin; Anthocyanins—Water-soluble pigments responsible for red, purple, and blue colors in plants.

**Table 2:** Representative examples of functional foods as vehicles of phytosterols and polyunsaturated fatty acids.

Food Source	Bioactive Compound(s)	Main Biological Action(s)	Reference
Vegetable oils (corn, soybean, rapeseed)	Phytosterols (β-sitosterol, campesterol, stigmasterol)	Competitive inhibition of intestinal cholesterol absorption; reduction of plasma LDL-C	[54]
Phytosterol-enriched margarine	Added plant sterols/stanols	Significant reduction in LDL-C in hypercholesterolemic subjects	[55]
Nuts (almonds, pistachios)	Phytosterols	LDL-C lowering; improvement of lipid profile; cardioprotective effects	[59]
Vegetable oils rich in ω-6 (sunflower, corn oil)	Linoleic acid (ω-6 PUFA)	Precursor of arachidonic acid and eicosanoids; modulation of inflammatory responses	[61]
Fatty fish (salmon, sardines, mackerel)	ω-3 PUFAs (EPA, DHA)	Anti-inflammatory activity; plaque stabilization; reduction in cardiovascular mortality	[63]
ω-3-enriched functional foods (dairy products, eggs, and bakery items)	EPA, DHA	Improvement of ω-6/ω-3 ratio; attenuation of chronic low-grade inflammation	[64]

Legend: LDL-C—Low-Density Lipoprotein Cholesterol; PUFA—Polyunsaturated Fatty Acid; ω-3/ω-6—Omega-3 and Omega-6 Fatty Acids; EPA—Eicosapentaenoic Acid; DHA—Docosahexaenoic Acid; Phytosterols—Plant-derived sterols structurally similar to cholesterol; Plant stanols—Saturated derivatives of plant sterols.

**Table 3:** Representative examples of functional foods as vehicles of vitamins.

Food Source	Bioactive Compound(s)	Main Biological Action(s)	Reference
Vegetable oils, nuts, seeds, whole grains	α-Tocopherol (Vitamin E)	Antioxidant, protects LDL-C from oxidation, reduces lipid peroxidation, improves endothelial function	[74]
Carrots, spinach, kale, peppers, apricots	β-Carotene, Lutein, Zeaxanthin (Vitamin A precursors)	Antioxidant, improves macular pigment density, reduces oxidative biomarkers, cardiovascular protection	[75]
Fatty fish, fortified dairy	Vitamin D_3_ (cholecalciferol)/Vitamin D_2_ (ergocalciferol)	Modulates oxidative stress, suppresses NADPH oxidase, enhances glutathione peroxidase, reduces pro-inflammatory cytokines	[76]
Leafy greens, broccoli, fermented foods	Vitamin K (phylloquinone, menaquinone)	Vascular protection, preserves NO bioavailability, reduces oxidative stress	[77]
Citrus fruits, kiwi, strawberries, peppers, berries	Vitamin C (ascorbic acid)	Radical scavenger, regenerates vitamin E, reduces oxidative stress and inflammation, improves endothelial function	[78]
Leafy greens, legumes, fortified grains	Folate (Vitamin B9)	Regulates homocysteine metabolism, reduces oxidative biomarkers, supports DNA synthesis	[79]
Meat, fish, poultry, eggs, dairy	Vitamin B12 (Cobalamin)	Cofactor for homocysteine metabolism, DNA synthesis, reduces oxidative stress indirectly	[80]
Meat, poultry, fish, legumes, nuts, cereals	Vitamin B6 (Pyridoxal-5′-phosphate)	Coenzyme in amino acid metabolism, reduces homocysteine, antioxidant cofactor	[81]
Whole grains, nuts, legumes, brewer’s yeast	Inositol (myo-inositol)	Modulates oxidative stress via Nrf2 and PI3K/Akt pathways, improves insulin sensitivity	[82]

Legend: LDL-C—Low-Density Lipoprotein Cholesterol; NO—Nitric Oxide; NADPH oxidase—Nicotinamide adenine dinucleotide phosphate oxidase; Nrf2—Nuclear factor erythroid 2-related factor 2; PI3K/Akt—Phosphatidylinositol 3-kinase/Protein kinase B signaling pathway; Vitamin D_3_—Cholecalciferol; Vitamin D_2_—Ergocalciferol; Myo-inositol—Vitamin-like cyclitol involved in insulin signaling.

**Table 4:** Representative functional foods modulating gut microbiota and associated health outcomes.

Food Source	Key Microbial Taxa Modulated	Main Health Outcomes	Reference
Kefir (milk-based)	*Bifidobacterium breve*, *Lactobacillus* spp., *Blautia*	Increased SCFA production (butyrate), improved gut barrier integrity, enhanced antioxidant status, immune modulation	[93]
Kombucha (tea-based)	*Lactobacillus*, *Weizmannia coagulans*, *Mucispirillum* spp.	Anti-inflammatory effects, improved liver metabolism, enhanced SCFA production	[95,96]
Miso (fermented soybean paste)	*Lactobacillus* spp., *Enterococcus* spp., *Bifidobacterium* spp.	Antioxidant activity, digestive enzyme support, immune modulation, cardiovascular protection	[97,98]
Sauerkraut (fermented cabbage)	*Lactobacillus plantarum*, *Leuconostoc* spp.	Increased vitamin C and K bioavailability, improved nutrient absorption, gut microbiota modulation	[99]
Synbiotic yogurt (probiotics + inulin/FOS)	*Lactobacillus rhamnosus*, *Bifidobacterium lactis*, *downstream SCFA-producing taxa*	Synergistic enhancement of SCFA production, improved metabolic profiles, gut barrier protection	[101]

Legend: SCFA(s)—Short-chain fatty acids (acetate, propionate, butyrate); spp.—Multiple species within the same genus; FOS—Fructooligosaccharides; Synbiotic—Combination of probiotics and prebiotics designed to act synergistically; Gut barrier integrity/function—Refers to maintenance of intestinal epithelial tight junctions and permeability control.

**Table 5:** Delivery challenges and advanced protection strategies for bioactive compounds in functional foods.

Bioactive Class	Stability & Bioavailability Challenges	Delivery/Protection Strategies	Reference
Polyphenols (EGCG, curcumin, anthocyanins, catechins, quercetin) Vitamins	Sensitive to heat, light, oxygen; prone to oxidation and polymerization; poor solubility in aqueous media; interactions with proteins reducing bioaccessibility	Nanoencapsulation in liposomes to enhance stability and intestinal delivery	[105]
Vitamin C	Sensitive to heat, light, oxygen; prone to oxidation and polymerization; poor solubility in aqueous media; interactions with proteins reducing bioaccessibility	Nanoencapsulation in liposomes to improve protection and controlled release	[106]
Curcumin	Lipophilic, low water solubility; sensitive to light, oxygen, heat; prone to isomerization	Nanoemulsions to enhance dispersibility, absorption, and stability	[109]
Omega-3 fatty acids & Lipophilic nutraceuticals (e.g., Vit D)	Oxidation, rancidity, low water solubility	Nanoemulsions, SLNs/NLCs, liposomes and co-encapsulation to improve oxidative stability and bioavailability	[110]
Polyphenols (e.g., chlorogenic acid, gallocatechin gallate)	Sensitive to heat, light, oxygen; prone to oxidation and polymerization; poor solubility in aqueous media; interactions with proteins reducing bioaccessibility	Protein–polysaccharide complexes to enhance protection and gastrointestinal delivery	[111]
Anthocyanins	Poor thermal stability	Gum Arabic-coated nanostructures to improve thermal stability and color retention	[112]
Polyphenols	Controlled release throughout gastrointestinal	Polysaccharide carriers (starch, maltodextrin, alginate, pectin, and inulin) for targeted and sustained release	[113]
Curcumin or lycopene	Poor solubility	Electrospinning and electrospraying to produce nano/micro-structured delivery systems	[114]
Valorized by-products (hazelnut skin, cereal bran, whey, fruit pomace)	Extraction efficiency; stability of recovered bioactives; taste/odor masking	NADES extraction, micro/nanoencapsulation and hydrogel incorporation to improve recovery, stability, and palatability	[115]

Legend: EGCG—Epigallocatechin-3-gallate; SLNs—Solid lipid nanoparticles; NLCs—Nanostructured lipid carriers; NADES—Natural deep eutectic solvents; Co-encapsulation—Simultaneous encapsulation of multiple bioactives in the same carrier system.

## Data Availability

No new data were created or analyzed in this study.

## References

[1] Fekete M., Lehoczki A., Kryczyk-Poprawa A., Zábó V., Varga J.T., Bálint M., Fazekas-Pongor V., Csípő T., Rząsa-Duran E., Varga P. (2025). Functional Foods in Modern Nutrition Science: Mechanisms, Evidence, and Public Health Implications. Nutrients.

[2] Skenderidou I., Leontopoulos S., Skenderidis P. (2025). Functional Food Ingredients Enhancing Immune Health: A Systematic Review. Int. J. Mol. Sci..

[3] Vignesh A., Amal T.C., Sarvalingam A., Vasanth K. (2024). A review on the influence of nutraceuticals and functional foods on health. Food Chem. Adv..

[4] Guo M., Guo M. (2009). Chapter 1—Introduction to functional foods. Functional Foods.

[5] Teodoro A.J. (2019). Bioactive Compounds of Food: Their Role in the Prevention and Treatment of Diseases. Oxidative Med. Cell. Longev..

[6] Shahidi F., Pan Y. (2022). Influence of food matrix and food processing on the chemical interaction and bioaccessibility of dietary phytochemicals: A review. Crit. Rev. Food Sci. Nutr..

[7] Kalogerakou T., Antoniadou M. (2024). The Role of Dietary Antioxidants, Food Supplements and Functional Foods for Energy Enhancement in Healthcare Professionals. Antioxidants.

[8] Selvaraj N.R., Nandan D., Nair B.G., Nair V.A., Venugopal P., Aradhya R. (2025). Oxidative Stress and Redox Imbalance: Common Mechanisms in Cancer Stem Cells and Neurodegenerative Diseases. Cells.

[9] Altanam S.Y., Darwish N., Bakillah A. (2025). Exploring the Interplay of Antioxidants, Inflammation, and Oxidative Stress: Mechanisms, Therapeutic Potential, and Clinical Implications. Diseases.

[10] Jyoti, Dey P. (2025). Mechanisms and implications of the gut microbial modulation of intestinal metabolic processes. npj Metab. Health Dis..

[11] Ballini A., Charitos I.A., Cantore S., Topi S., Bottalico L., Santacroce L. (2023). About Functional Foods: The Probiotics and Prebiotics State of Art. Antibiotics.

[12] Marangoni F., Poli A. (2010). Phytosterols and cardiovascular health. Pharm. Res..

[13] Li H., Cao W., Tao L., Zhu Y. (2025). The role of fat-soluble vitamins on bone metabolism and osteoporosis: A literature review. Ann. Med..

[14] Talib W.H., Ahmed Jum’AH D.A., Attallah Z.S., Jallad M.S., Al Kury L.T., Hadi R.W., Mahmod A.I. (2024). Role of vitamins A, C, D, E in cancer prevention and therapy: Therapeutic potentials and mechanisms of action. Front. Nutr..

[15] Rein M.J., Renouf M., Cruz-Hernandez C., Actis-Goretta L., Thakkar S.K., da Silva Pinto M. (2013). Bioavailability of bioactive food compounds: A challenging journey to bioefficacy. Br. J. Clin. Pharmacol..

[16] Garcia-Llorens G., El Ouardi M., Valls-Belles V. (2025). Oxidative Stress Fundamentals: Unraveling the Pathophysiological Role of Redox Imbalance in Non-Communicable Diseases. Appl. Sci..

[17] Chandimali N., Bak S.G., Park E.H., Lim H.J., Won Y.S., Kim E.K., Park S.I., Lee S.J. (2025). Free radicals and their impact on health and antioxidant defenses: A review. Cell Death Discov..

[18] Chen Y.S., Tian H.X., Rong D.C., Wang L., Chen S., Zeng J., Xu H., Mei J., Wang L.Y., Liou Y.L. (2025). ROS homeostasis in cell fate, pathophysiology, and therapeutic interventions. Mol. Biomed..

[19] Liu S., Liu J., Wang Y., Deng F., Deng Z. (2025). Oxidative Stress: Signaling Pathways, Biological Functions, and Disease. MedComm.

[20] Turrens J.F. (2003). Mitochondrial formation of reactive oxygen species. J. Physiol..

[21] Canton M., Sánchez-Rodríguez R., Spera I., Venegas F.C., Favia M., Viola A., Castegna A. (2021). Reactive Oxygen Species in Macrophages: Sources and Targets. Front. Immunol..

[22] Iacopetta D., Ceramella J., Catalano A., Scali E., Scumaci D., Pellegrino M., Aquaro S., Saturnino C., Sinicropi M.S. (2023). Impact of Cytochrome P450 Enzymes on the Phase I Metabolism of Drugs. Appl. Sci..

[23] Vázquez-Galán Y.I., Guzmán-Silahua S., Trujillo-Rangel W.Á., Rodríguez-Lara S.Q. (2025). Role of Ischemia/Reperfusion and Oxidative Stress in Shock State. Cells.

[24] Zheng F., Gonçalves F.M., Abiko Y., Li H., Kumagai Y., Aschner M. (2020). Redox toxicology of environmental chemicals causing oxidative stress. Redox Biol..

[25] Wang Y., Branicky R., Noë A., Hekimi S. (2018). Superoxide dismutases: Dual roles in controlling ROS damage and regulating ROS signaling. J. Cell Biol..

[26] Mondola P., Damiano S., Sasso A., Santillo M. (2016). The Cu, Zn Superoxide Dismutase: Not Only a Dismutase Enzyme. Front. Physiol..

[27] Rasheed Z. (2024). Therapeutic potentials of catalase: Mechanisms, applications, and future perspectives. Int. J. Health Sci..

[28] Orian L., Flohé L. (2021). Selenium-Catalyzed Reduction of Hydroperoxides in Chemistry and Biology. Antioxidants.

[29] Jomova K., Alomar S.Y., Valko R., Nepovimova E., Kuca K., Valko M. (2025). The role of redox-active iron, copper, manganese, and redox-inactive zinc in toxicity, oxidative stress, and human diseases. EXCLI J..

[30] Asad S.F., Singh S., Ahmad A., Khan N., Hadi S.M. (2001). Prooxidant and antioxidant activities of bilirubin and its metabolic precursor biliverdin: A structure-activity study. Chem.-Biol. Interact..

[31] Albuja-Quintana N., Chisaguano-Tonato A.M., Herrera-Fontana M.E., Figueroa-Samaniego S., Alvarez-Suarez J.M. (2025). Relationship between plasma uric acid levels, antioxidant capacity, and oxidative damage markers in overweight and obese adults: A cross-sectional study. PLoS ONE.

[32] Grosso G. (2018). Effects of Polyphenol-Rich Foods on Human Health. Nutrients.

[33] Potì F., Santi D., Spaggiari G., Zimetti F., Zanotti I. (2019). Polyphenol Health Effects on Cardiovascular and Neurodegenerative Disorders: A Review and Meta-Analysis. Int. J. Mol. Sci..

[34] Fekete M., Jarecsny T., Lehoczki A., Major D., Fazekas-Pongor V., Csípő T., Lipécz Á., Szappanos Á., Pázmándi E.M., Varga P. (2025). Mediterranean Diet, Polyphenols, and Neuroprotection: Mechanistic Insights into Resveratrol and Oleuropein. Nutrients.

[35] Medina-Remón A., Casas R., Tresserra-Rimbau A., Ros E., Martínez-González M., Fitó M., Corella D., Salas-Salvadó J., Lamuela-Raventós R.M., Estruch R. (2016). Polyphenol intake from a Mediterranean diet decreases inflammatory biomarkers related to atherosclerosis: A sub-study of The PREDIMED trial. Br. J. Clin. Pharmacol..

[36] Lachowicz-Wiśniewska S., Świeca M., Kapusta I., Sip A., Ochmian I. (2025). Comparative assessment of polyphenol stability, bioactivity, and digestive availability in purified compounds and fruit extracts from four black chokeberry cultivars. Sci. Rep..

[37] Bohn T. (2014). Dietary factors affecting polyphenol bioavailability. Nutr. Rev..

[38] Obeme-Nmom J.I., Abioye R.O., Reyes Flores S.S., Udenigwe C.C. (2024). Regulation of redox enzymes by nutraceuticals: A review of the roles of antioxidant polyphenols and peptides. Food Funct..

[39] Popiolek-Kalisz J., Fornal E. (2022). The Impact of Flavonols on Cardiovascular Risk. Nutrients.

[40] Lee K.H., Park E., Lee H.J., Kim M.O., Cha Y.J., Kim J.M., Lee H., Shin M.J. (2011). Effects of daily quercetin-rich supplementation on cardiometabolic risks in male smokers. Nutr. Res. Pract..

[41] Grassi D., Desideri G., Necozione S., di Giosia P., Barnabei R., Allegaert L., Bernaert H., Ferri C. (2015). Cocoa consumption dose-dependently improves flow-mediated dilation and arterial stiffness decreasing blood pressure in healthy individuals. J. Hypertens..

[42] Thielecke F., Boschmann M. (2009). The potential role of green tea catechins in the prevention of the metabolic syndrome—A review. Phytochemistry.

[43] Castro-Acosta M., Lenihan-Geels G., Corpe C., Hall W. (2016). Berries and anthocyanins: Promising functional food ingredients with postprandial glycaemia-lowering effects. Proc. Nutr. Soc..

[44] Ahles S., Joris P.J., Plat J. (2021). Effects of Berry Anthocyanins on Cognitive Performance, Vascular Function and Cardiometabolic Risk Markers: A Systematic Review of Randomized Placebo-Controlled Intervention Studies in Humans. Int. J. Mol. Sci..

[45] Ryan-Borchers T.A., Park J.S., Chew B.P., McGuire M.K., Fournier L.R., Beerman K.A. (2006). Soy isoflavones modulate immune function in healthy postmenopausal women^2^. Am. J. Clin. Nutr..

[46] Adetunji J.A., Fasae K.D., Awe A.I., Paimo O.K., Adegoke A.M., Akintunde J.K., Sekhoacha M.P. (2023). The protective roles of citrus flavonoids, naringenin, and naringin on endothelial cell dysfunction in diseases. Heliyon.

[47] Zhang Y., Zhang Q., Wang X., Jia Y., Niu Q., Ding S., Li W. (2025). Effects of phytosterol-rich foods on lipid profile and inflammatory markers in patients with hyperlipidemia: A systematic review and meta-analysis. Front. Pharmacol..

[48] Kupikowska-Stobba B., Niu H., Klojdová I., Agregán R., Lorenzo J.M., Kasprzak M. (2025). Controlled lipid digestion in the development of functional and personalized foods for a tailored delivery of dietary fats. Food Chem..

[49] Ruscica M., Loh W.J., Sirtori C.R., Watts G.F. (2025). Phytosterols and phytostanols in context: From physiology and pathophysiology to food supplementation and clinical practice. Pharmacol. Res..

[50] Gutierres D., Pacheco R., Reis C.P. (2025). The Role of Omega-3 and Omega-6 Polyunsaturated Fatty Acid Supplementation in Human Health. Foods.

[51] Dyall S.C., Balas L., Bazan N.G., Brenna J.T., Chiang N., da Costa Souza F., Dalli J., Durand T., Galano J.M., Lein P.J. (2022). Polyunsaturated fatty acids and fatty acid-derived lipid mediators: Recent advances in the understanding of their biosynthesis, structures, and functions. Prog. Lipid Res..

[52] Lund-Blix N.A., Rønningen K.S., Bøås H., Tapia G., Andersen L.F. (2016). Plasma phospholipid pentadecanoic acid, EPA, and DHA, and the frequency of dairy and fish product intake in young children. Food Nutr. Res..

[53] Maestre R., Douglass J.D., Kodukula S., Medina I., Storch J. (2013). Alterations in the Intestinal Assimilation of Oxidized PUFAs Are Ameliorated by a Polyphenol-Rich Grape Seed Extract in an In Vitro Model and Caco-2 Cells. J. Nutr..

[54] Li X., Xin Y., Mo Y., Marozik P., He T., Guo H. (2022). The Bioavailability and Biological Activities of Phytosterols as Modulators of Cholesterol Metabolism. Molecules.

[55] Mannarino E., Pirro M., Cortese C., Lupattelli G., Siepi D., Mezzetti A., Bertolini S., Parillo M., Fellin R., Pujia A. (2008). Effects of a phytosterol-enriched dairy product on lipids, sterols and 8-isoprostane in hypercholesterolemic patients: A multicenter Italian study. Nutr. Metab. Cardiovasc. Dis. NMCD.

[56] Teupser D., Baber R., Ceglarek U., Scholz M., Illig T., Gieger C., Holdt L.M., Leichtle A., Greiser K.H., Huster D. (2010). Genetic Regulation of Serum Phytosterol Levels and Risk of Coronary Artery Disease. Circ. Cardiovasc. Genet..

[57] Ganesan R., Henkels K.M., Wrenshall L.E., Kanaho Y., Di Paolo G., Frohman M.A., Gomez-Cambronero J. (2018). Oxidized LDL phagocytosis during foam cell formation in atherosclerotic plaques relies on a PLD2-CD36 functional interdependence. J. Leukoc. Biol..

[58] Ros E. (2010). Health benefits of nut consumption. Nutrients.

[59] Ros E., Singh A., O’Keefe J. (2021). Nuts: Natural Pleiotropic Nutraceuticals. Nutrients.

[60] Jackson K.H., Harris W.S., Belury M.A., Kris-Etherton P.M., Calder P.C. (2024). Beneficial effects of linoleic acid on cardiometabolic health: An update. Lipids Health Dis..

[61] Mohammadi-Nasrabadi F., Alhouei B., Khoshtinat K., Rashidimehr A., Esfarjani F. (2025). Fatty Acid Quality in Sunflower and Frying Oils: Implications for Nutritional Health. J. Food Qual..

[62] Patterson E., Wall R., Fitzgerald G.F., Ross R.P., Stanton C. (2012). Health Implications of High Dietary Omega-6 Polyunsaturated Fatty Acids. J. Nutr. Metab..

[63] Tsoupras A., Brummell C., Kealy C., Vitkaitis K., Redfern S., Zabetakis I. (2022). Cardio-Protective Properties and Health Benefits of Fish Lipid Bioactives; The Effects of Thermal Processing. Mar. Drugs.

[64] Patel A., Desai S.S., Mane V.K., Enman J., Rova U., Christakopoulos P., Matsakas L. (2022). Futuristic food fortification with a balanced ratio of dietary ω-3/ω-6 omega fatty acids for the prevention of lifestyle diseases. Trends Food Sci. Technol..

[65] Fang M., Xiang C., Zhang L., Li P. (2025). Effect Differences of Omega-3 Fatty Acids From Plant Oil and Fish Oil on Human Health. AgriFood.

[66] Padalkar P., Zende P., Fedotova J.O. (2025). Vitamins in Health and Diseases. Vitamins and Human Health.

[67] Jia H., Ren F., Liu H. (2024). Evaluation of bioaccessibility and bioavailability of dietary bioactives and their application in food systems. Food Biosci..

[68] Andrès E., Lorenzo-Villalba N., Terrade J.E., Méndez-Bailon M. (2024). Fat-Soluble Vitamins A, D, E, and K: Review of the Literature and Points of Interest for the Clinician. J. Clin. Med..

[69] Yang Y., Decker E., Xiao H., McClements D. (2014). Enhancing vitamin E bioaccessibility: Factors impacting solubilization and hydrolysis of α-tocopherol acetate encapsulated in emulsion-based delivery systems. Food Funct..

[70] Dawson-Hughes B., Harris S.S., Lichtenstein A.H., Dolnikowski G., Palermo N.J., Rasmussen H. (2015). Dietary Fat Increases Vitamin D-3 Absorption. J. Acad. Nutr. Diet..

[71] Geng Y., Cui K., Ding N., Liu H., Huo J., Sui X., Zhang Y. (2024). Polyphenol co-pigments enhanced the antioxidant capacity and color stability of blue honeysuckle juice during storage. Food Chem. X.

[72] Bermúdez-Humarán L.G., Chassaing B., Langella P. (2024). Exploring the interaction and impact of probiotic and commensal bacteria on vitamins, minerals and short chain fatty acids metabolism. Microb. Cell Factories.

[73] McMillan D.C., Maguire D., Talwar D. (2019). Relationship between nutritional status and the systemic inflammatory response: Micronutrients. Proc. Nutr. Soc..

[74] Jiang Q. (2014). Natural forms of vitamin E: Metabolism, antioxidant, and anti-inflammatory activities and their role in disease prevention and therapy. Free Radic. Biol. Med..

[75] Yamagata K., Cvetković D.J., Nikolic G.S. (2017). Carotenoids Regulate Endothelial Functions and Reduce the Risk of Cardiovascular Disease. Carotenoids.

[76] Fenercioglu A.K. (2024). The Anti-Inflammatory Roles of Vitamin D for Improving Human Health. Curr. Issues Mol. Biol..

[77] Sim M., Lewis J.R., Prince R.L., Levinger I., Brennan-Speranza T.C., Palmer C., Bondonno C.P., Bondonno N.P., Devine A., Ward N.C. (2020). The effects of vitamin K-rich green leafy vegetables on bone metabolism: A 4-week randomised controlled trial in middle-aged and older individuals. Bone Rep..

[78] Ashor A.W., Lara J., Mathers J.C., Siervo M. (2014). Effect of vitamin C on endothelial function in health and disease: A systematic review and meta-analysis of randomised controlled trials. Atherosclerosis.

[79] Pravst I., Lavriša Ž., Hribar M., Hristov H., Kvarantan N., Seljak B.K., Gregorič M., Blaznik U., Gregorič N., Zaletel K. (2021). Dietary Intake of Folate and Assessment of the Folate Deficiency Prevalence in Slovenia Using Serum Biomarkers. Nutrients.

[80] Obeid R., Heil S.G., Verhoeven M.M.A., van den Heuvel E., de Groot L., Eussen S. (2019). Vitamin B12 Intake From Animal Foods, Biomarkers, and Health Aspects. Front. Nutr..

[81] Santos A., Khemiri S., Simões S., Prista C., Sousa I., Raymundo A. (2023). The importance, prevalence and determination of vitamins B6 and B12 in food matrices: A review. Food Chem..

[82] Caputo M., Bona E., Leone I., Samà M.T., Nuzzo A., Ferrero A., Aimaretti G., Marzullo P., Prodam F. (2020). Inositols and metabolic disorders: From farm to bedside. J. Tradit. Complement. Med..

[83] Ogunrinola G.A., Oyewale J.O., Oshamika O.O., Olasehinde G.I. (2020). The Human Microbiome and Its Impacts on Health. Int. J. Microbiol..

[84] Kumar S., Mukherjee R., Gaur P., Leal É., Lyu X., Ahmad S., Puri P., Chang C.-M., Raj V.S., Pandey R.P. (2025). Unveiling roles of beneficial gut bacteria and optimal diets for health. Front. Microbiol..

[85] Du Y., He C., An Y., Huang Y., Zhang H., Fu W., Wang M., Shan Z., Xie J., Yang Y. (2024). The Role of Short Chain Fatty Acids in Inflammation and Body Health. Int. J. Mol. Sci..

[86] Mahanta S., Nath K., Boruah K., Chakraborty T. (2026). Dysbiosis and the modern lifestyle: Mechanisms, health impacts, and microbiome based interventions. Brain Behav. Immun. Integr..

[87] Victoria Obayomi O., Folakemi Olaniran A., Olugbemiga Owa S. (2024). Unveiling the role of functional foods with emphasis on prebiotics and probiotics in human health: A review. J. Funct. Foods.

[88] Aguilera J. (2018). The Food Matrix: Implications in Processing, Nutrition and Health. Crit. Rev. Food Sci. Nutr..

[89] Sankarganesh P., Bhunia A., Ganesh Kumar A., Babu A.S., Gopukumar S.T., Lokesh E. (2025). Short-chain fatty acids (SCFAs) in gut health: Implications for drug metabolism and therapeutics. Med. Microecol..

[90] van der Hee B., Wells J.M. (2021). Microbial Regulation of Host Physiology by Short-chain Fatty Acids. Trends Microbiol..

[91] Van-Wehle T., Vital M. (2024). Investigating the response of the butyrate production potential to major fibers in dietary intervention studies. NPJ Biofilms Microbiomes.

[92] Kosmerl E., González-Orozco B.D., García-Cano I., Ortega-Anaya J., Jiménez-Flores R. (2023). Milk phospholipids protect Bifidobacterium longum subsp. infantis during in vitro digestion and enhance polysaccharide production. Front. Nutr..

[93] Choi Y., Keum G.B., Kang J., Doo H., Kwak J., Kim H., Chae Y., Lee S., Yang H., Kim S. (2025). Evaluation of kefir consumption on gut microbial diversity in a healthy young population using full-length 16S rRNA sequencing. Front. Microbiol..

[94] Yılmaz İ., Dolar M.E., Özpınar H. (2019). Effect of administering kefir on the changes in fecal microbiota and symptoms of inflammatory bowel disease: A randomized controlled trial. Turk. J. Gastroenterol. Off. J. Turk. Soc. Gastroenterol..

[95] Jung Y., Kim I., Mannaa M., Kim J., Wang S., Park I., Kim J., Seo Y.-S. (2018). Effect of Kombucha on gut-microbiota in mouse having non-alcoholic fatty liver disease. Food Sci. Biotechnol..

[96] Fraiz G.M., Bonifácio D.B., de Paulo R.S., Teixeira C.M., Martino H.S.D., Barros F.A.R.d., Milagro F.I., Bressan J. (2025). Benefits of Kombucha Consumption: A Systematic Review of Clinical Trials Focused on Microbiota and Metabolic Health. Fermentation.

[97] Saeed F., Afzaal M., Shah Y.A., Khan M.H., Hussain M., Ikram A., Ateeq H., Noman M., Saewan S.A., Khashroum A.O. (2022). Miso: A traditional nutritious & health-endorsing fermented product. Food Sci. Nutr..

[98] Hashimoto Y., Okamura T., Bamba R., Yoshimura Y., Munekawa C., Kaji A., Miki A., Majima S., Senmaru T., Ushigome E. (2024). Miso, fermented soybean paste, suppresses high-fat/high-sucrose diet-induced muscle atrophy in mice. J. Clin. Biochem. Nutr..

[99] Wei L., Marco M. (2025). The fermented cabbage metabolome and its protection against cytokine-induced intestinal barrier disruption of Caco-2 monolayers. Appl. Environ. Microbiol..

[100] Rastall R., Gibson G. (2015). Recent developments in prebiotics to selectively impact beneficial microbes and promote intestinal health. Curr. Opin. Biotechnol..

[101] Miller B., Mainali R., Nagpal R., Yadav H. (2021). A Newly Developed Synbiotic Yogurt Prevents Diabetes by Improving the Microbiome–Intestine–Pancreas Axis. Int. J. Mol. Sci..

[102] Zhang J., Shadrack S.M., Wang M., Mi S., Chu M., Rakariyatham K., McClements D.J., Cao C., Xu X., Yuan B. (2026). Overcoming oral delivery barriers of functional proteins: Current status and advanced delivery technologies. Food Chem..

[103] Bravo-Núñez Á., Valéro R., Reboul E. (2025). Evaluating the roles of food matrix, lipid micronutrients and bioactives in controlling postprandial hypertriglyceridaemia and inflammation. Nutr. Res. Rev..

[104] Taouzinet L., Djaoudene O., Fatmi S., Bouiche C., Amrane-Abider M., Bougherra H., Rezgui F., Madani K. (2023). Trends of Nanoencapsulation Strategy for Natural Compounds in the Food Industry. Processes.

[105] Popovici V., Boldianu A.B., Pintea A., Caraus V., Ghendov-Mosanu A., Subotin I., Druta R., Sturza R. (2024). In Vitro Antioxidant Activity of Liposomal Formulations of Sea Buckthorn and Grape Pomace. Foods.

[106] Dhotre T., Thanawala S., Shah R. (2025). Optimizing Oral Vitamin C Supplementation: Addressing Pharmacokinetic Challenges with Nutraceutical Formulation Approaches—A Mini Review. Pharmaceutics.

[107] Liu Q., Huang H., Chen H., Lin J., Wang Q. (2019). Food-Grade Nanoemulsions: Preparation, Stability and Application in Encapsulation of Bioactive Compounds. Molecules.

[108] Hao M., Tan X., Liu K., Xin N. (2026). Nanoencapsulation of nutraceuticals: Enhancing stability and bioavailability in functional foods. Front. Nutr..

[109] Ochoa-Flores A.A., Hernández-Becerra J.A., Cavazos-Garduño A., Soto-Rodríguez I., Sanchez-Otero M.G., Vernon-Carter E.J., García H.S. (2017). Enhanced Bioavailability of Curcumin Nanoemulsions Stabilized with Phosphatidylcholine Modified with Medium Chain Fatty Acids. Curr. Drug Deliv..

[110] Shakeri M., Ghobadi R., Sohrabvandi S., Khanniri E., Mollakhalili-Meybodi N. (2024). Co-encapsulation of omega-3 and vitamin D_3_ in beeswax solid lipid nanoparticles to evaluate physicochemical and in vitro release properties. Front. Nutr..

[111] Sahraeian S., Rashidinejad A., Golmakani M.-T. (2024). Recent advances in the conjugation approaches for enhancing the bioavailability of polyphenols. Food Hydrocoll..

[112] Guan Y., Zhong Q. (2015). The improved thermal stability of anthocyanins at pH 5.0 by gum arabic. LWT-Food Sci. Technol..

[113] Shuaibu A., Juma N.S., Bashir B.H. (2026). Polysaccharides as Carriers and Protectors of Additives and Bioactive Compounds in Food. J. Future Foods.

[114] Ghorbani M., Aleman R.S., Queiroz Zepka L., Casagrande Do Nascimento T., Jacob-Lopes E. (2021). Electro-Spinning and Electro-Spraying as Innovative Approaches in Developing of a Suitable Food Vehicle for Polyphenols-Based Functional Ingredients. Bioactive Compounds—Biosynthesis, Characterization and Applications.

[115] Conte R., Sepe F., Margarucci S., Costanzo E., Petillo O., Peluso G., Marcolongo L., Calarco A. (2025). Functional Plant-Based Beverage Fortified with Hazelnut Cuticle Polyphenols: Antioxidant and Phenolic Content Characterization. Molecules.

